# A comparison of anxiety and depression between pre-dialysis chronic kidney disease patients and hemodialysis patients using hospital anxiety and depression scale

**DOI:** 10.12669/pjms.334.12656

**Published:** 2017

**Authors:** Salman T. Shafi, Tahir Shafi

**Affiliations:** 1 Salman T. Shafi, Department of Nephrology, Sharif Medical and Dental College, Sharif Medical City Road Jati Umra, Lahore, Pakistan; 2 Tahir Shafi, Department of Nephrology, Sharif Medical and Dental College, Sharif Medical City Road Jati Umra, Lahore, Pakistan

**Keywords:** Anxiety, Depression, Chronic kidney disease, Hemodialysis, Comparison

## Abstract

**Objective:**

Tocompare frequency of anxiety and depression between pre-dialysis chronic kidney disease (CKD) and hemodialysis patients (ESRD) in Pakistan.

**Methods:**

This study was conducted in an out-patient department and hemodialysis unit of Sharif Medical City Hospital. Inclusion criteria included age above 18 years and a diagnosis of CKD including both pre-dialysis CKD and ESRD patients. Patients were screened for anxiety and depression using hospital anxiety and depression scale (HADS).

**Results:**

A total of 156 patients were included in the study. Out of these patients, 81 (51.9%) had ESRD and 75 (48.1%) had pre-dialysis CKD. Mean age of all patient was 47.3±18.3 years, 96 (61.5%) were males and 60 (38.5%) were females. Median duration of renal disease was 16 months (IQR 8-36 months). Anxiety and depression were present in 111 (71.2%) and 113 (72.4%) of all patients respectively. Moderate to severe anxiety and depression were present in 54 (34.6%) and 60 (38.5%) patients respectively. In multiple logistic regression model, after adjusting for other variables, ESRD vs. pre-dialysis CKD was significantly associated with moderate to severe depression (AOR 2.26 (1.1-5.1).

**Conclusion:**

Both anxiety and depression are common in pre-dialysis CKD and ESRD patients. Patients with ESRD have higher frequency of depression compared to pre-dialysis CKD patients.

## INTRODUCTION

Chronic kidney disease (CKD) is a growing public health problem.^1^ Depression is common in patients with CKD and is associated with adverse outcomes.^2^ In a meta-analysis and systematic review of observational studies, depression was found in 22.8% (confidence interval (CI), 18.6-27.6) of dialysis patientsand 21.4% of patients with pre-dialysis CKD (CI, 11.1-37.2) based on clinical structured interview (CSI). Using self- or clinician-administered rating scales, the prevalence of depression was found to behigher (39.3% (CI, 36.8-42.0)) in dialysis patients relative to CKD stages 1-5 (26.5% (CI, 18.5-36.5)).^3^ In Pakistan, prevalence of depression has been found to be higher in various studies, focused mainly on hemodialysis patients. The reported prevalence of depression in hemodialysis patients is 48.8%-83.8%,^4^–^8^ whereas a single study has reported prevalence of depression in pre-dialysis CKD patients as 61.1%.^5^

Though less studied compared to depression, anxiety is also common in CKD patients.^9^ Prevalence of anxiety has been found to be 21.1%-53.4% in hemodialysis and pre-dialysis CKD patients.^10^–^13^ In local studies, anxiety was present in 34.9%-42.7% of hemodialysis patients.^6^,^7^

The local literature on frequency of anxiety and depression is mainly focused on hemodialysis patients. There is limited local data on comparison of both anxiety and depression between pre-dialysis CKD and hemodialysis patients. The objective of this study was to compare frequency of anxiety and depression between pre-dialysis CKD and ESRD patients.

## METHODS

This study was conducted in an out-patient department and hemodialysis unit of a department of nephrology at Sharif Medical City Hospital over a one month period. The study was approved by institutional ethics committee. Patients were included in the study after obtaining informed consent from them. Inclusion criteria included age above 18 years and a diagnosis of CKD including patients with end stage renal disease (ESRD) on hemodialysis. Both pre-dialysis CKD and hemodialysis patients were included. Pre-dialysis CKDwas defined as estimated glomerular filtration rate (eGFR) of less than <60ml/min/1.73m^2^ or presence of proteinuria for three or more months,^14^ but not on any renal replacement therapy. eGFR was calculated by CKD-EPI formula.^15^ ESRD was defined as patients undergoing hemodialysis for more than four weeks.

Patient’s history, medical records and laboratory information were reviewed to obtain data on their age, sex, height, weight, address, educational level, socio-economic, marital, employment, smoking status, duration of renal disease, hypertension, diabetes mellitus, cardiovascular disease, hepatitis C, blood hemoglobin, serum calcium, phosphorous, albumin and serum creatinine.

Weight was measured using a manual scale with accuracy up to 0.5kg. Height was measured on barefoot patients using a fixed stadiometer with the measurement taken to the nearest 0.1 cm. Body mass index (BMI) was calculated as weight in kilograms divided by height in meter square. A patient was considered to be hypertensive or diabeticif he or she had been diagnosed by a health care provider and/or is receiving treatment for either disease Cardiovascular disease was defined as known prior history of coronary artery disease, congestive heart failure, peripheral vascular disease or cerebrovascular disease based on history and review of prior medical records. A low income class was defined as daily income of less than twice the upper limit of accepted poverty line i-e $2 per household person. A smoker was defined according to categories from the US Centers for Disease Control and Prevention, with current smokers being those adults who have smoked 100 cigarettes in their lifetime and currently smoke cigarettes every day (daily) or some days (nondaily). Serum creatinine was measured by Jaffe method using a kit by AMP Diagnostics, Austria.

Anxiety and depression were assessed using hospital anxiety and depression scale (HADS) which was filled out by clinician.^16^ This scale is a questionnaire that consists of 14 individual items. Out of 14 items, 7 items evaluate anxiety and the remaining items assess depression. Total score for each anxiety and depression is between 0 and 21. A score of 0-7 is considered normal. Mild, moderate and severe grades are assigned for scores of 8-10, 11-14 and 15-21 respectively. For our study, we used threshold score of 8 or above for anxiety and depression and score of 11 or higher for “moderate to severe” anxiety and depression.

### Statistical Analysis

Continuous parametric variables were reported as means ± standard deviation and non-parametric continuous variables were reported as median with 25–75 inter quartile range. Categorical variables were reported as percentages. Categorical variables were compared using the chi-square test. Student’s t test was used to compare continuous variables. Pearson correlation was used to evaluate association between anxiety and depression scores and eGFR in patients with pre-dialysis CKD. Multiple logistic regression analysis was used to study unadjusted and adjusted association between predictor variables including ESRD vs. pre-dialysis CKD and moderate to severe anxiety and depression. Due to relatively small sample size, we included only demographic variables and variables with an unadjusted p value of 0.10 or less in the final model. All statistical analyses were performed using SPSS 20.0 (Chicago, IL USA). For all tests, p value of <0.05 was considered statistically significant.

## RESULTS

A total of 156 patients were included in the study. Out of these patients, 81 (51.9%) had ESRD and 75 (48.1%) had pre-dialysisCKD. Mean age of all patients was 47.3±18.3 years, 96 (61.5%) were males and 60 (38.5%) were females. Median duration of renal disease was 16 months (IQR 8-36 months). Hypertension, diabetes mellitus and cardiovascular disease were present in 82.1%, 47.4% and 19.9% of all patients respectively. Mean anxiety score was 9.2±3.6. Mean depression score was 9.1±3.9. Anxiety and depression were present in 111 (71.2%) and 113 (72.4%) of all patients respectively. Moderate to severe anxiety and depression were present in 34.6% and 38.7% of all patients respectively.

A comparison of characteristics of patients with pre-dialysis CKD and ESRD is shown in Table-I. Patients with pre-dialysis CKD were older, more likely to be married, diabetic and resident of urban area and had better socioeconomic status.

**Table-I:** A comparison of characteristics of patients with Pre-dialysis CKD and ESRD.

	*Chronic Kidney Disease not on hemodialysis (Predialysis CKD) N=75*	*End Stage Renal Disease on Hemodialysis (ESRD) N=81*	*P value*
Mean age in years	54.8±16.5	40.4±17.3	<0.01
Male sex (%)	56.8	66.7	0.20
Married (%)	89.2	71.6	<0.01
Urban Address (%)	57.3	42.7	0.05
Middle or high socioeconomic status (%)	50.0	33.8	0.04
Secondary or higher education (%)	47.3	48.1	0.92
Working (%)	42.6	57.4	0.36
Smoker (%)	10.8	6.2	0.29
Median duration of renal disease in months (IQR)	12 (6-24)	18 (9-36)	0.12
Hypertension (%)	84.0	80.2	0.54
Diabetes Mellitus (%)	62.2	37.8	<0.01
Cardiovascular Disease (%)	23	17.9	0.44
Median Body mass index (Kg/m^2^)	21.7 (16.9-31.9)	18.9 (15.6-23.4)	0.50

Comparison of anxiety and depression scores between pre-dialysis CKD and ESRD patients is shown in Table-II. Patients with ESRD had higher mean depression scores and higher frequency of depression compared to pre-dialysis CKD patients.

**Table-II:** Comparison of Anxiety and Depression scores between Pre-dialysis CKD and ESRD patients.

	*Chronic Kidney Disease not on Hemodialysis (Predialysis CKD) N=75*	*End Stage Renal Disease on Hemodialysis (ESRD) N=81*	*P value*
Mean anxiety Scores	9.4±3.8	9.1±3.3	0.66
Mean depression Scores	8.2±3.8	9.9±3.8	<0.01
Patients with anxiety (%)	68	70.4	0.75
Patients with moderate to severe anxiety (%)	37.3	32.1	0.49
Patients with depression (%)	64.9	80.2	0.03
Patients with moderate to severe depression (%)	27	49.4	<0.01

Multiple logistic regression model of association of predictor variables including ESRD vs. pre-dialysis CKD with moderate to severe depression is shown in Table-III. In the final model, ESRD vs. pre-dialysis CKD was positively while residence in urban area was negatively associated with moderate to severe depression.

**Table-III:** Multivariate logistic regression model of predictors (including ESRD vs. CKD) of moderate to severe depression.

*Variables*	*B*	*Standard Error*	*P value*	*Adjusted Odds ratio*	*95% Confidence interval*
Age > 50 years	-0.47	0.42	0.27	0.63	0.27-1.43
Male Sex	-0.78	0.42	0.06	0.46	0.20-1.05
Urban Address	-1.29	0.45	<0.01	0.28	0.16-0.66
Middle or higher socioeconomic status	-0.39	0.45	0.39	0.68	0.28-1.64
ESRD vs. pre dialysis CKD	0.81	0.41	0.04	2.26	1.1-5.1
Diabetes Mellitus	-0.51	0.41	0.21	0.68	0.27-1.34

There was no significant association between any predictor variables including ESRD vs. CKD and moderate to severe anxiety in univariate or in multivariate logistic regression model.

In patients with pre-dialysis CKD, median eGFR was 17.6 ml/min/1.73 m^2^ (IQR 7.8-24.5). Of these patients, 14.8% had stage III, 42.6% had stage IV and 42.6% had stage V CKD. There was no significant co-relation between eGFR and anxiety score (r= -0.02, p value 0.87) or eGFR and depression score (r= -0.07, p value 0.58).

Only age, sex, ESRD vs. pre-dialysis CKD and variables with p<0.10 on univariate analysis were included in the final model.

## DISCUSSION

In our study, we found that anxiety and depression were present in 71.2% and 72.4% of all CKD patients including patients on hemodialysis. Frequency of moderate to severe anxiety was similar in pre-dialysis CKD and ESRD patients, but ESRD patients have higher frequency of moderate to severe depression compared to pre-dialysis CKD patients after adjusting for other variables.

Frequency of depression in our study is somewhat comparable to other local studies. Depression was found in 75-83.8% of hemodialysis patients.^4^,^5^,^8^ However in some studies, frequency of depression was found to be lower (48.8-57.3%).^6^,^7^ Frequency of depression in pre-dialysis CKD patients in our study was 64%, which is comparable to that ofstudy by Nomani et al.^5^ We also showed in our study that compared to pre-dialysis CKD, ESRD is associated with higher depression score after adjusting for other variables. In a study by Nomaniet al, which evaluated frequency of depression in patients between pre-dialysis CKD and ESRD, adjustment for other confounding variables was not made.^5^ Frequency of depression in our pre-dialysis CKD and ESRD patients is higher compared to international data, though results are comparable if only frequency of moderate to severe depression is considered (39.3% in ESRD and 26.5% in pre-dialysis CKD by self or clinician administered rating scales).^3^ Though not specifically elucidated in our study, higher prevalence of depression in our patient population may be related to socio-economic factors and perception of somatic symptoms as manifestations of depression by self or clinician administered rating scales.

In our study, we found that residence in urban area was inversely associated with moderate to severe depression. In local studies, factors associated with depression in hemodialysis patients were found to be male sex,^7^ marriage,^7^,^8^ low income status,^7^,^8^ number of children,^8^ un-employment,^8^ anemia^4^ and lower educational level.^5^,^8^ In international studies, factors associated with depression in hemodialysis patients were found to be age,^13^,^17^ family problems,^13^ female sex,^12^,^17^,^18^ white race,^17^,^19^ poor sleep quality,^18^ unemploymentduration of dialysis, diabetes,^17^–^19^ cardiovascular disease,^19^ hypoalbuminemia,^18^ low education,^18^ pruritus,^18^ alcohol use^12^ anduse of erythropoietin.^12^. Whereas in CKD patients, factors associated with depression included lack of religious belief, lack of exerciseand severity of CKD.^20^ Our study population has both pre-dialysis CKD and hemodialysis patients which could explain variation in results. Other factors include difference in sample size, assessment tools to evaluate depression and lack of multivariate analysis to adjust for confounders in other studies.

Frequency of anxiety was present in 71.2% of patients with no significant difference between pre-dialysis CKD and ESRD patients. Moderate to severe anxiety was found in 34.6% of all patients. High frequency of anxiety in pre-dialysis CKD patients in our patient population has not been previously demonstrated. Frequency of anxiety in our study population is higher compared to other studies, though results are somewhat comparable if only frequency of moderate to severe anxiety is considered. In Turkey, prevalence of anxiety was found to be 53.4% in pre-dialysis CKD patients.^10^ InUS hemodialysis patients, anxiety was found by structured interview in 27%-45.7% of patients,^11^,^21^ whereas in China and Saudi Arabia , anxiety was found in 36.9%^12^ and 21.1%^13^ in hemodialysis patients. In local studies, anxiety was present in 34.9%-42.7% of hemodialysis patients by HADS scale.^6^,^7^ There is limited local and international data on factors associated with anxiety in CKD patients. In a study by Hou et al. female sex and alcohol usewere associated with anxiety in hemodialysis patients.^12^

High prevalence of depression and anxiety in our patient population is a significant finding. Depression is associated with increased mortality, hospitalization rates, poor treatment compliance, poor quality of life in patients with CKD including those not on hemodialysis.^22^,^23^ Depression has also been associated with faster decline in kidney function in pre-dialysis CKD patients.^23^ Anxiety in pre-dialysis CKD patients is associated with poor clinical outcome.^24^

### Limitations of the study

It is a single center study with somewhat limited sample size. We used a rating scale to screen anxiety and depression in our patient population rather than structured clinical interview by a qualified psychiatrist. Rating scales may over-estimate diagnosis of depression in CKD patients.^3^ However, both HADS and Beck’s depressioninventory scales (BDI) have been found to be valid in diagnosis of depression and anxiety in ESRD patients.^25^

In summary, frequency of anxiety and depression is high in pre-dialysis CKD patients and in patients on hemodialysis. Hemodialysis patients have higher frequency of depression compared to pre-dialysis CKD patients, while there is no difference in anxiety between pre-dialysis CKD and ESRD patients. Clinicians should routinely assess both pre-dialysis and ESRD patients for anxiety and depression. Further studies are needed to determine impact of anxiety and depression on clinical outcomes in our patient population.

### Authors’ Contributions

***Salman T. Shafi:*** Study design, literature review, assigning people for data collection, data analysis and manuscript writing.

***TahirShafi:*** Manuscript writing and review.

***Funding:*** None.

***Disclosure:*** No Conflict of interest for all authors.
